# Longitudinal changes in bodyweight, body condition, and muscle condition in ageing pet cats: findings from the Cat Prospective Ageing and Welfare Study

**DOI:** 10.3389/fvets.2025.1654002

**Published:** 2025-08-25

**Authors:** Christine R. Pye, Nathalie J. Dowgray, Kelly Eyre, Gina Pinchbeck, Vincent Biourge, Delphine Moniot, Eithne Comerford, Alexander J. German

**Affiliations:** ^1^Department of Musculoskeletal Biology and Ageing Science, Institute of Life Course and Medical Sciences, University of Liverpool, Liverpool, United Kingdom; ^2^Department of Livestock and One Health, Institute of Infection, Veterinary and Ecological Studies, University of Liverpool, Liverpool, United Kingdom; ^3^Royal Canin Research Centre, Aimargues, France; ^4^Department of Small Animal Clinical Science, School of Veterinary Sciences, University of Liverpool, Liverpool, United Kingdom

**Keywords:** ageing, cats, body composition, muscle, bodyweight, longitudinal, sex differences, morbidity

## Abstract

Body composition metrics such as bodyweight, body condition score (BCS) and muscle condition score (MCS) can be readily recorded as part of veterinary examinations in ageing cats. However, the description of how these parameters change with age, whilst accounting for sex and age-related morbidity, is limited. The aim of this prospective cohort study was to evaluate age, sex and health-related changes in bodyweight, BCS and MCS in client-owned pet cats. A total of 1,231 veterinary examinations were performed on 209 pet cats aged 6.7–16.4 years enrolled on the Cat Prospective Ageing and Welfare Study. Cats were followed every 6 months for up to 7 years. Mixed-effects models using natural cubic splines were applied to investigate non-linear age-related changes, adjusting for sex and disease status. All three metrics showed significant non-linear associations with age. Bodyweight increased slightly from age 7 to 10 (estimated marginal mean 4.77–4.82 kg) before decreasing to 4.30 kg by age 16 years. Body condition score also increased slightly during middle age, before declining after age 10.5 years. The combined MCS (maximum 30 points, based on 10 skeletal landmarks) decreased gradually from 30 points to 28 points between ages 7 and 10, before decreasing at a greater rate to 15 points by age 16. After age 10, the cats had a greater probability of mild muscle loss than no muscle loss, and by age 14, they were more likely to be underweight than obese. Male cats were heavier and had 2.8 times greater odds of being overweight (*p* = 0.002); there were no sex differences in MCS. Cats that developed chronic disease had a greater age-related decline in all three metrics. Associations with various chronic diseases were identified, including a positive association between orthopaedic abnormalities and bodyweight. These findings indicate that muscle loss begins before middle age in cats, whilst loss of body condition and bodyweight begins after age 10. The results also outline the importance of monitoring body composition metrics in veterinary examinations of ageing cats.

## 1 Introduction

The median lifespan of cats attending UK primary care practices has been estimated at 14 years (1), although this lifespan does not necessarily correspond with ‘healthspan’, defined as the length of time living in good health (2). Cats enter the ‘mature’ life stage at 7 years of age, and the ‘senior’ life stage at >10 years of age (3). As in humans, advancing age is a risk factor for morbidity in cats, including cardiovascular disease, cancer, chronic kidney disease (CKD), endocrinopathies and musculoskeletal disorders (4–7). Body composition also changes with age in cats, with a loss of lean body mass in senior cats (8). Such changes are associated with underlying physiological mechanisms of the ageing process (9), as well as being affected by (and potentially increasing the risk of) certain age-related diseases (10–13). In clinical practice, metrics such as bodyweight, body condition score (BCS) and muscle condition score (MCS) can be routinely measured and provide insights into body composition, without the need for specialist equipment (14, 15). Understanding how such metrics change as cats age, whilst accounting for possible effects of sex and chronic disease, could help to identify targets for improving quality of life in ageing cats, for example, through interventions such as diet, environmental modifications and pharmaceutical treatments.

Age-related changes in bodyweight and BCS in cats have been investigated in cross-sectional studies (16–18), although such studies are limited by the fact that the rate of ageing varies amongst individual cats due to genetics, environment, diet and disease (19, 20). Longitudinal studies of ageing have advantages over cross-sectional studies because they enable changes in physiological biomarkers to be assessed over time both within and between subjects. For example, changes in body composition in ageing cats living in research colonies have been examined in longitudinal studies, although these studies have not accounted for differences in sex (8, 21, 22). Retrospective data from electronic health records have been used to assess longitudinal changes in bodyweight and sex with age in cats, but not accounting for health status (23). Longitudinal studies have also been used to examine alterations in body composition resulting from chronic diseases, including hyperthyroidism (12), CKD (24) and heart failure (25).

Therefore, there is currently a gap in the literature regarding prospective longitudinal age, sex and health related changes in body composition metrics in client-owned pet cats. Findings from mature cats (aged 7 to 10 years) at enrolment to a longitudinal study of ageing in a client-owned cat cohort have recently been reported (26). The objectives of the current study were to use data collected from the same cohort of client-owned pet cats followed from middle age onwards for up to 7 years to determine longitudinal changes in bodyweight, BCS and MCS with ageing, whilst adjusting for the effects of sex and illness.

## 2 Materials and methods

### 2.1 Study design and ethical approval

This was a prospective, longitudinal study of ageing in client-owned pet cats [the Cat Prospective Ageing and Welfare Study (CatPAWS) (26)]. A brief overview of design and study methodology is provided below, with the full details already reported elsewhere (26, 27). Ethical approval was granted by the University of Liverpool Veterinary Research Ethics Committee, under the code VREC491 (with amendments abcde), and the Royal Canin Ethical Review Committee. All owners gave informed, written consent for their cat’s participation.

### 2.2 Data collection

Data were collected from cats who were first enrolled aged 6.7 to 10 years between February 2017 and March 2020. Follow-up visits were scheduled every 6 months thereafter, until death or censoring or until the time of review for this study (January 2024). At each visit, a thorough clinical history was obtained using owner questionnaires (to ascertain changes in the cats’ behaviour and activity) and additional non-scripted questioning as required. A physical examination was conducted by either a veterinarian (yearly in cats <10 years and biannually in cats >10 years) or a registered veterinary nurse (at interim 6-monthly appointments for cats <10 years). Any evidence of lameness was recorded, and a complete orthopaedic examination (OE) was conducted on amenable cats (26, 28). Blood samples were collected, by jugular venepuncture, for serum biochemical and haematological analysis, whilst systolic blood pressure was measured using the Doppler method (Thames medical CAT+ Doppler or Vet BP Doppler; cuff size 2.5–3.0 cm) according to standard guidelines (29). The systolic blood pressure, serum biochemistry and haematology tests were used to diagnose associated diseases, although specific results were not addressed in the current study.

### 2.3 Body composition metrics

**Figure 1:**
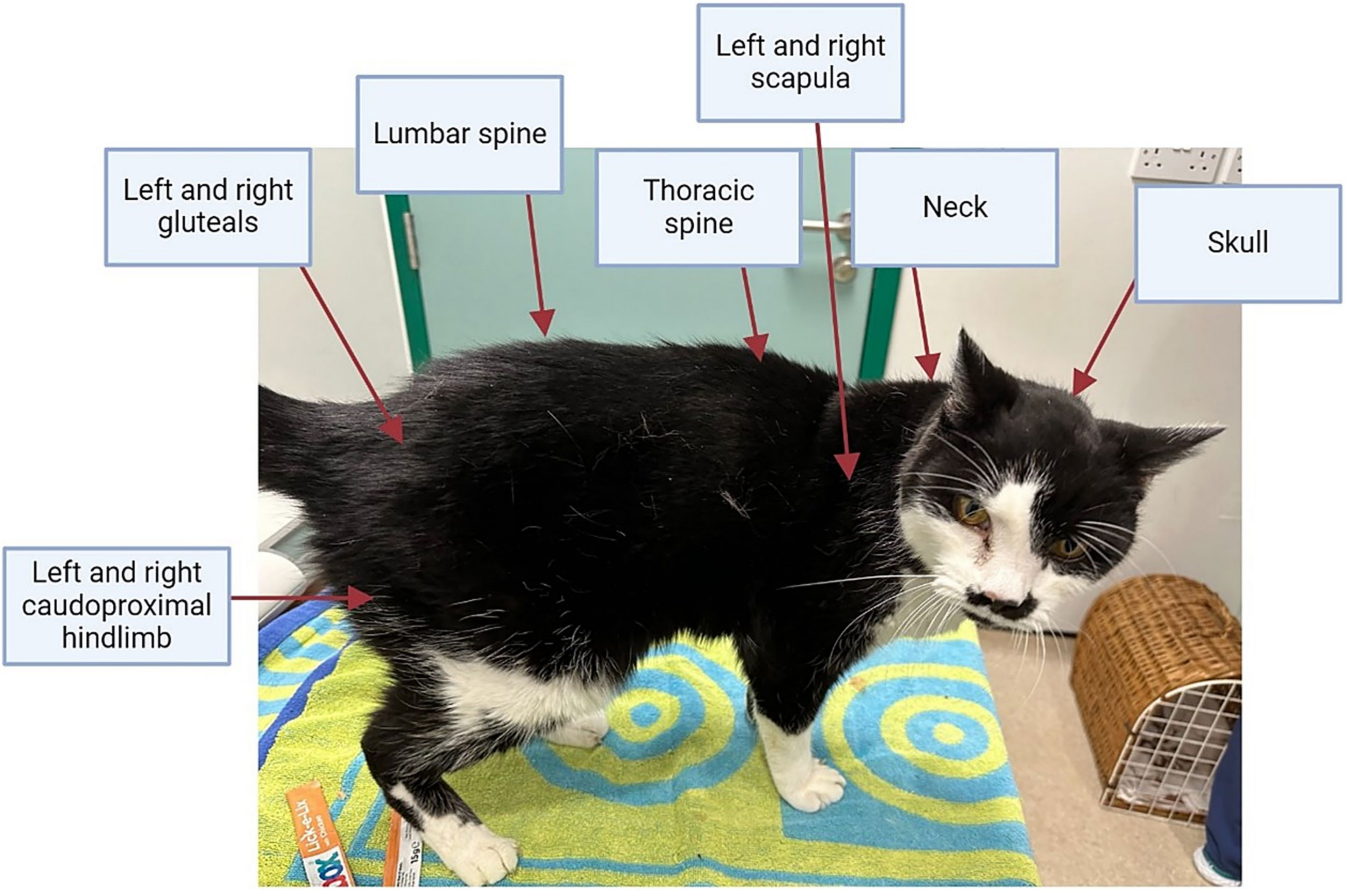
Skeletal landmarks palpated to ascertain the combined muscle condition score in cats. Each of the 10 skeletal landmarks were graded from 3 (no muscle loss) to 0 (severe muscle loss), giving a combined muscle condition score of up to 30 points.

At each examination, bodyweight was measured using portable V20 feline scales (Burtons, United Kingdom), which were regularly calibrated with a ‘test’ weight, whilst BCS was assessed using a 9-unit scale, as previously described (14). Muscle condition score was assessed at 10 separate skeletal landmarks: skull, neck, thoracic vertebrae, lumbar vertebrae, left and right scapula, left and right gluteal muscle group and left and right hindlimb muscle groups caudal to the femur (Figure 1). Each skeletal landmark was graded from 0 (severe muscle wastage) to 3 (no muscle wastage) using a validated scale (15), with a combined MCS score up to a maximum of 30 points created by summing results from all 10 landmarks.

### 2.4 Classification of health and disease status

For each visit, the presence of any chronic diseases and abnormal clinical findings were recorded, including CKD, hyperthyroidism, hypertension, neoplasia, heart murmurs, diabetes mellitus and dental disease. Diagnostic criteria for such diseases and clinical findings have been reported previously (26–28). The presence or absence of abnormalities on OE was also recorded, except for cats which did not tolerate an OE, which were instead recorded as ‘unable to perform OE’ (26–28).

To investigate the possible impact of chronic disease on body composition metrics, cats were assigned a health status of “healthy” or “not healthy” at each visit. Cats were defined as “healthy” if they fulfilled the following criteria: (1) absence of hypertension, CKD, hyperthyroidism, a heart murmur ≥ grade 3, arrythmia or diagnosed cardiac disease, neoplasia, diabetes mellitus or cognitive dysfunction; (2) absence of other chronic diseases that might plausibly affect body composition [e.g., gastrointestinal (GI) disease, chronic cat flu, feline immunodeficiency virus (FIV)]; (3) absence of a severe acute systemic disease at time of sampling (e.g., infectious disease, pancreatitis, liver disease); (4) no signs of orthopaedic disease or severe dental disease. Finally, an “overall health status” was assigned for each cat across the study period. Cats that did not die or develop any chronic disease by the time of study completion or censoring were classified as ageing healthily. All other cats were considered not to be ageing healthily overall.

### 2.5 Statistical analysis

All statistical analysis was performed using an open-access statistical language and environment [R version 4.4.1; (30)]. The following body composition metrics were examined: bodyweight, BCS and combined MCS. Normality of these metrics were examined using histograms, Q-Q plots and Shapiro–Wilk testing, and metrics were described using either mean and standard deviation (SD) or median and interquartile range (IQR), depending on whether or not their distribution was gaussian.

Linear mixed-effects models, with restricted maximum likelihood, were used to investigate changes in body composition metrics with age. The packages in R used for these analyses were ‘lme4’ version 1.1–35.4 (31) and ‘splines’ [version 4.4.1; (30)], whilst graphs and tables were created with ‘ggplot2’ version 3.5.1 (32) and ‘sjPlot’ version 2.8.16 (33).

For bodyweight, BCS and combined MCS, the following research questions were asked:

(1) How does the body composition metric change with increasing age, accounting for potential non-linear relationships between age and the body composition metric, and does this change differ by sex?
(2) How does the body composition metric change with increasing age, accounting for potential non-linear relationships between age and the body composition metric, and does this change differ by both sex and health status over the course of the study?
(3) How does the body composition metric change with increasing age, accounting for potential non-linear relationships between age and body composition metric, and how does this relationship differ according to different disease states?

To answer these questions, three separate linear mixed-effects models were developed for each body composition metric as indicated below:

Model 1. Fixed effects of age, sex, and the interaction between age and sex; individual cat included as a random effect.
Model 2. Fixed effects of age, sex and the interaction of age and sex, overall health status and the interactions of overall health status and age; individual cat included as a random effect.
Model 3. Fixed effects of age, sex, and diagnosis of CKD, hyperthyroidism, hypertension, neoplasia, heart murmurs, diabetes mellitus and dental disease plus whether OE abnormalities were present; individual cat included as a random effect.

The R code for these models are given in the Supplementary Table 1.

Breed was not included as a covariate in the models because the majority of cats in the cohort were of mixed breed (180/209, 86%), whilst the remaining 14% (29 cats) represented 14 different pedigree breeds. The sample sizes for individual breeds were therefore too small to allow meaningful statistical comparisons. Additionally, only two cats were of a large-framed breed (Maine Coons: one male and one female) with an even distribution of age and sex.

Natural cubic splines were included in all models to investigate possible non-linear effects of age (34, 35). The degrees of freedom used were selected based on investigations of the generalisability of models which used different degrees of freedom (from 2 to 5), and selecting the model of best fit using log likelihood ratio test, Akaike Information Criterion (AIC) and Bayesian Information Criterion (BIC). The position of the internal knot for natural cubic splines applied to age in two degrees of freedom was determined by the 50th percentile of the age data distribution, which was marginally greater than 10 years of age. The ‘check_model’ function of the ‘performance’ package [version 0.12.0; (36)] was used to assess model performance, with specific checks including a posterior predictive check (to compare the distribution of data for the outcome variable with predictions from the model), homogeneity of variance, influential observations, multicollinearity (by analysing variance inflation factors), normality of residuals and normality of random effects. In addition, autocorrelation was assessed by plotting autocorrelation function plots. Estimated marginal means (EMMs) were obtained from the linear mixed-effects models by the ‘emmeans’ package [version 1.10.2; (37)].

Multinomial mixed-effects models were created to investigate changes in categorical variables with age, sex and health status using the ‘mblogit’ function of the ‘mclogit’ package version 0.9.6 (38). Body condition score was separated into four categories: underweight (BCS 1–3/9), ideal weight (BCS 4–5/9), overweight (BCS 6–7/9) and obese (BCS 8–9/9). The combined MCS, derived from the 10 skeletal landmarks, was originally categorised into no muscle loss (MCS 30/30), mild muscle loss (MCS 20–29/30), moderate muscle loss (MCS 10–19/30) and severe muscle loss (MCS 0–9/30). However, given that only one cat had a combined MCS classed as severe, this category was subsequently amalgamated with the moderate muscle loss category to create a combined moderate-to-severe muscle loss category (MCS 0–19/30). The resulting 4-unit variable (for BCS) and 3-unit variable (for the combined MCS) were modelled using cat age (again using natural cubic splines with 2 degrees of freedom), sex and overall health status; interactions amongst these variables were added to models if they improved the fit (based on log likelihood ratio test and AIC). Plots of the predicted effects of these models were visualised using ggplot2, with generalised additive model (GAM) or locally estimated scatterplot smoothing (LOESS) applied depending on the default ‘geom_smooth’ function setting (32).

Body condition score and the combined MCS data were therefore analysed as both continuous variables using linear mixed-effects models and as categorical variables using multinomial mixed-effects models. Treating these variables as continuous enabled the assessment of overall age-related trajectories using spline-based modeling and allowed comparison with bodyweight. Although BCS and MCS are technically ordinal, their scale (9-point for BCS; 30-point for combined MCS) supports approximation as continuous measures for modeling purposes (39–41). Multinomial models were then also employed to assess age-related changes in grouped BCS and MCS categories. This dual approach allowed for both visual interpretation of non-linear age-related trajectories and consideration of categories of body condition and muscle condition.

To account for multiple testing, the Benjamini–Hochberg false discovery rate (FDR) correction was applied to the *p*-values associated with the fixed effects in each model. Significance was set as FDR adjusted *p*-value of <0.05.

## 3 Results

### 3.1 Summary of study population and body composition data

A summary of the available observations of cats and their bodyweight, BCS and combined MCS are shown in Table 1. Sample sizes differed amongst body composition metrics depending on whether measurement of the metric during a particular examination had been possible as a result of the temperament of the cat. Most data were available for bodyweight (median 6 per cat, IQR 3–9, range 1–12) and BCS (median 6 per cat, IQR 3–9, range 1–12), with fewer observations available for MCS (median 5, IQR 2–8, range 1–12). There were 98 male and 111 female cats, with all but 2 (both males with a total of 4 observations from age 7.0–8.3 years) being neutered. Median age was 10.3 years (IQR 8.8–12.0, range 6.7–16.4 years). Twenty-nine of the 209 cats (14%) were pedigrees, with 14 different breeds represented (Supplementary Table 2), whilst the remaining cats (180, 86%) were from non-pedigree breeds. In total, 59/209 cats (28%) died over the study period. There was a relatively even distribution of sex and health status across the age range studied. However, cats that remained healthy over time tended to be slightly younger than those that developed morbidity (healthy cats: median 9.6 years, IQR 8.2–11.0; not healthy: median 10.6 years, IQR 9.1–12.3; Table 1). Distributions of age by sex, health status, and breed are shown in Supplementary Figure 1.

**Table 1:** An overview of the total sample sizes for each body composition metric recorded in cats enrolled on the Cat Prospective Ageing and Welfare Study.

Dependent variable	Total		Sex^1^		Overall health status^2^		Enrolment^3^	Age^4^
	Obs^5^	Cats	Obs^5^	Cats	Obs^5^	Cats		
Bodyweight (kg)	1,231	209	587/644 (48/52%)	98/111 (47/53%)	321/910 (26/74%)	73/136 (35/65%)	4.65 (3.98–5.39)	Female: 10.3 (8.7–11.9; 6.7–16.0) Male: 10.4 (9.0–12.0; 6.9–16.4) Healthy: 9.6 (8.2–11; 6.9–15.4) Not healthy: 10.6 (9.1–12.3; 6.7–16.4)
Body condition ^6^	1,220	209	585/628 (48/52%)	98/111 (47/53%)	314/906 (26/74%)	73/136 (35/65%)	6 (5–7)	Female: 10.3 (8.8–11.9; 6.7–16.0) Male: 10.4 (9.0–12.0; 6.9–16.4) Healthy: 9.6 (8.2–11; 6.9–15.4) Not healthy: 10.6 (9.1–12.3; 6.7–16.4)
Muscle condition ^7^	1,144	208	554/590 (48/52%)	97/111 (47/53%)	287/857 (25/75%)	72/136 (35/65%)	30 (28–30)	Female: 10.2 (8.7–11.9; 6.7–16.0) Male: 10.4 (9.0–12.0; 6.9–16.4) Healthy: 9.5 (8.2–10.9; 6.9–15.4) Not healthy: 10.6 (9.1–12.3; 6.7–16.4)

^1^Sex reported as number (%) of cats (male / female). ^2^Overall health status over the study period is the presence (“not healthy”) or absence (“healthy”) of chronic diseases or death that each cat developed over the study period with results reported as number (%) of cats (healthy/not healthy). ^3^Results of the dependent variable at enrolment to the study reported as median (inter-quartile range). ^4^Age is given in years, presented as median (IQR; range). ^5^Obs: observations. ^6^Body condition assessed using a 9-unit scale (14). ^7^Muscle condition is the combined score for 10 separate landmarks, with each graded from 0 (severe muscle wastage) to 3 (no muscle wastage) using a validated scale (15).

The sample sizes for variables used in models 1 and 2 are provided in Table 1. For model 3, which included various disease states, the sample sizes are shown in Table 2. For each model, only complete cases with all variables recorded were included, therefore sample sizes were smaller in model 3 than those used in models 1 and 2 because of removal of missing values from certain covariates where data were not available.

**Table 2:** An overview of the total sample sizes for each body composition metric where details on various morbidity diagnoses were also available in cats enrolled on the Cat Prospective Ageing and Welfare Study.

Dependent variable	Total		Sex^1^		CKD^2^		Hyperthyroid^2^		Hypertension^2^		Neoplasia^2^		Heart murmur^2^		Diabetes mellitus^2^		Dental disease^2^		OE abnormalities^3^	
	Obs^4^	Cats	Obs^4^	Cats	Obs^4^	Cats	Obs^4^	Cats	Obs^4^	Cats	Obs^4^	Cats	Obs^4^	Cats	Obs^4^	Cats	Obs^4^	Cats	Obs^4^	Cats
Bodyweight (kg)	1,055	203	525/530 (50/50%)	98/105 (48/52%)	90 (9%)	22 (11%)	64 (6%)	18 (9%)	139 (13%)	52 (26%)	9 (1%)	8 (4%)	320 (30%)	122 (60%)	9 (1%)	4 (2%)	599 (57%)	170 (84%)	124/331/600 (18/31/ 57%)	75/156/169 (37/77/57%)
Body condition^5^	1,052	203	524/528 (50/50%)	98/105 (48/52%)	90 (9%)	22 (11%)	64 (6%)	18 (9%)	139 (13%)	52 (26%)	9 (1%)	8 (4%)	320 (30%)	122 (60%)	9 (1%)	4 (2%)	598 (57%)	170 (84%)	124/328/600 (18/31/ 57%)	75/154/169 (37/76/57%)
Muscle condition^6^	1,013	202	507/506 (50/50%)	98/104 (49/51%)	88 (9%)	22 (11%)	63 (6%)	18 (9%)	136 (13%)	52 (26%)	9 (1%)	8 (4%)	313 (31%)	122 (60%)	7 (1%)	4 (2%)	578 (57%)	170 (84%)	121/303/589 (18/30/ 58%)	75/154/169 (37/76/58%)

^1^Sex reported as number (%) of cats (male / female). ^2^Results reported as number (%) of cats with diagnosis. ^3^Orthopaedic examination (OE) abnormalities reported as either no OE abnormalities (“No”), OE not performed or OE abnormalities present (“Yes”). Results reported as number (%) of cats (No/OE not performed/Yes). ^4^Obs: observations. ^5^Body condition assessed using a 9-unit scale (14). ^6^Muscle condition is the combined score for 10 separate landmarks, with each graded from 0 (severe muscle wastage) to 3 (no muscle wastage) using a validated scale (15). CKD, chronic kidney disease; OE, orthopaedic examination. The sample sizes shown in this table were used in multivariate linear mixed effects models where all morbidity diagnoses were included as covariates.

### 3.2 Bodyweight

**Figure 2:**
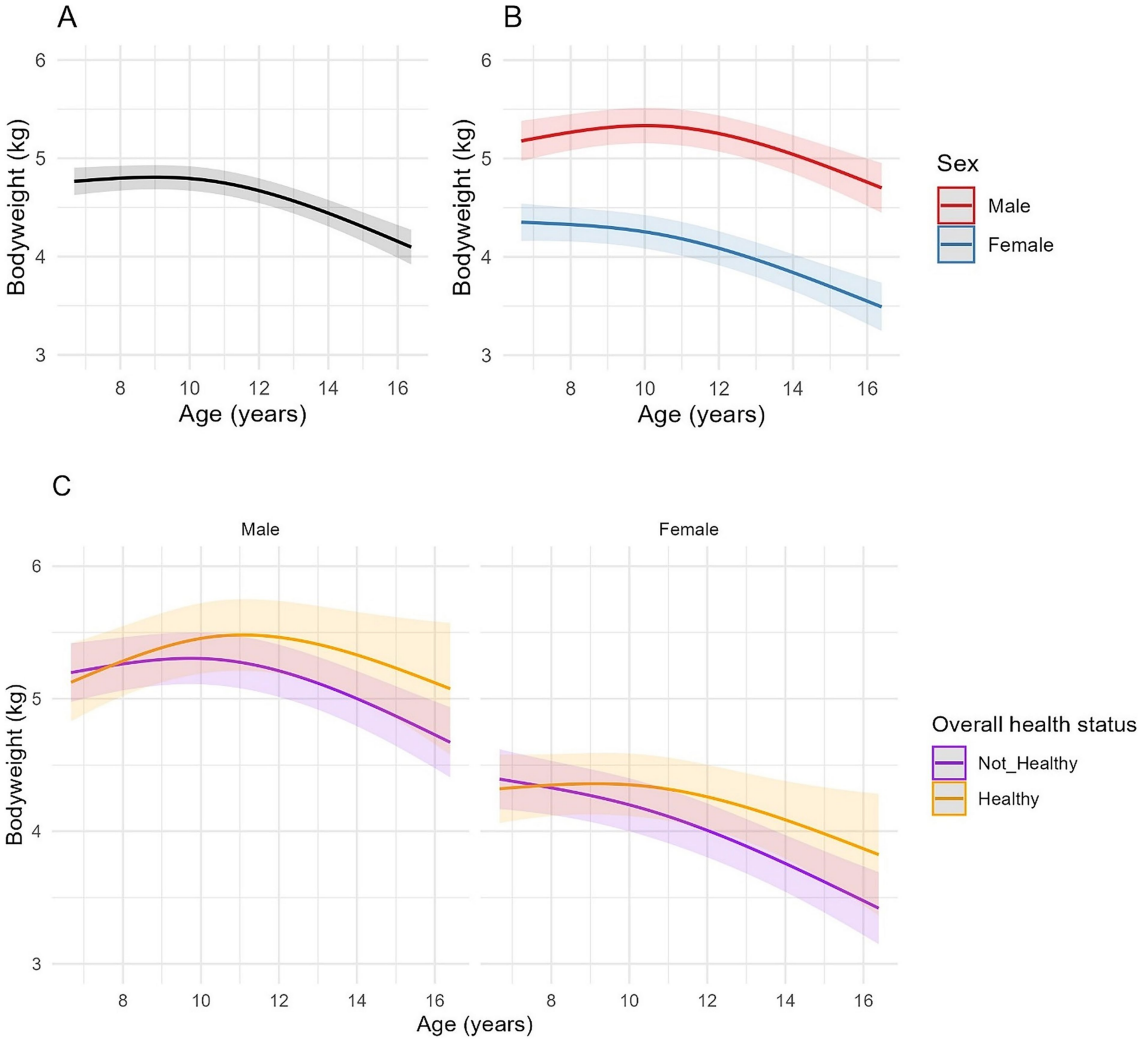
Longitudinal changes in bodyweight in cats enrolled on the Cat Prospective Ageing and Welfare Study. **(A,B)** Show the outcome of linear mixed-effects models with fixed effects of age (modelled with natural cubic splines for age in two degrees of freedom to account for non-linear associations of bodyweight and age), sex and the interactions of age and sex from 1,231 veterinary examinations from 209 cats, whilst **(C)** also included the overall health status of the cat over the course of the study and its interactions with age. The individual cat was added as a random effect. **(A)** Overall changes in bodyweight for all cats; **(B)** Overall changes in bodyweight for all cats separated by sex; **(C)** Overall changes in bodyweight in cats separated by sex (male cats shown in the left-side plot, female cats in the right-side plot) and whether the cat remained healthy over the study period or developed chronic morbidities and/or died (classed as “Not healthy”).

Bodyweight measurements were recorded at 1,231 visits for 209 cats (Table 1). Median bodyweight at enrolment was 4.65 kg (IQR 3.98–5.39 kg). Spaghetti plots, displaying longitudinal trends within cats, are included in the Supplementary Figure 2. The outcomes of the three linear mixed-effects models investigating changes in bodyweight in the cats with age, sex and health-status are shown in Table 3 and Figure 2. There was substantial variability in bodyweight amongst cats, with the variance of the random intercept being 0.78–0.80. The intraclass correlation coefficient (ICC) was large (0.86–0.87), indicating that ~86–87% of the variance in bodyweight is due to the differences amongst cats. The fit of all three models was good, with 90–91% of the variance in bodyweight being explained by the combination of fixed and random effects.

**Table 3:** Outcomes of linear mixed-effects models with natural cubic splines for age examining the effects of age, sex, overall health status, and morbidities on bodyweight in 209 cats enrolled on the Cat Prospective Ageing and Welfare Study.

Predictors	Bodyweight (kg) [model 1]				Bodyweight (kg) [model 2]				Bodyweight (kg) [model 3]			
	Estimates	CI	Raw *p*-value	FDR adjusted *p*-value	Estimates	CI	Raw *p*-value	FDR adjusted *p*-value	Estimates	CI	Raw *p*-value	FDR adjusted *p*-value
(Intercept)	4.35	4.16–4.54	**<0.001**	**<0.001**	4.32	4.06–4.58	**<0.001**	**<0.001**	4.25	4.04–4.46	**<0.001**	**<0.001**
Age (years) [1st degree]	−0.60	−0.82 – −0.38	**<0.001**	**<0.001**	−0.20	−0.51 – 0.12	0.226	0.254	−0.60	−0.85 – −0.35	**<0.001**	**<0.001**
Age (years) [2nd degree]	−0.83	−1.02 – −0.65	**<0.001**	**<0.001**	−0.54	−0.95 – −0.14	**0.009**	0.016	−0.66	−0.87 – −0.45	**<0.001**	**<0.001**
Sex [Male v Female at baseline]	0.83	0.55–1.10	**<0.001**	**<0.001**	0.80	0.52–1.08	**<0.001**	**<0.001**	0.80	0.52–1.09	**<0.001**	**<0.001**
Age (years) [1st degree] x Sex [Male]	0.62	0.30–0.95	**<0.001**	**<0.001**	0.74	0.41–1.06	**<0.001**	**<0.001**	0.74	0.40–1.09	**<0.001**	**<0.001**
Age (years) [2nd degree] x Sex [Male]	0.25	−0.01 – 0.52	0.063	0.063	0.29	0.02–0.56	**0.034**	0.051	0.18	−0.10 – 0.47	0.213	0.246
Health status over study [Not Healthy vs Healthy at baseline]					0.07	−0.22 – 0.37	0.630	0.630				
Age (years) [1st degree] x Health status over study [Not Healthy]					−0.63	−0.99 – −0.27	**0.001**	**0.002**				
Age (years) [2nd degree] x Health status over study [Not Healthy]					−0.34	−0.77 – 0.08	0.115	0.147				
CKD									−0.21	−0.38 – −0.05	**0.013**	**0.019**
Hyperthyroidism									−0.40	−0.55 – −0.25	**<0.001**	**<0.001**
Hypertension									−0.02	−0.10 – 0.07	0.729	0.729
Neoplasia									−0.40	−0.67 – −0.14	**0.003**	**0.005**
Heart murmur									−0.03	−0.10 – 0.03	0.273	0.292
Diabetes mellitus									−0.35	−0.68 – −0.02	**0.039**	0.054
Dental disease									0.04	−0.01 – 0.09	0.144	0.180
OE abnormalities [Unable to perform OE]									0.16	0.07–0.25	**<0.001**	**<0.001**
OE abnormalities [Yes]									0.15	0.07–0.24	**<0.001**	**<0.001**
	Random effects											
σ^2^	0.12				0.12				0.12			
τ_00_	0.78 _Cat_ID_				0.78 _Cat_ID_				0.81 _Cat_ID_			
ICC	0.86				0.87				0.87			
*N*	209 _Cat_ID_				209 _Cat_ID_				203 _Cat_ID_			
Observations	1,231				1,231				1,055			
Marginal *R*^2^/Conditional *R*^2^	0.252/0.899				0.258/0.901				0.263/0.907			

Age (years) 1st and 2nd degree relate to the 1st and 2nd degrees of freedom for a natural cubic spline model considering non-linear associations of age. Health status over study relates to whether the cat aged “healthily” or “not healthily” over the study period, i.e., whether the cat had developed morbidity or mortality by the end of the study period. *p*-values were adjusted for false discovery rate using the Benjamini-Hochberg method. CI, 95% confidence intervals; CKD, chronic kidney disease; OE, orthopaedic examination; ICC, intraclass correlation; FDR, false discovery rate. Values in bold indicate raw and FDR adjusted *p*-values of <0.05.

A non-linear association between age and bodyweight was identified in all models (Table 3, Figure 2). There was also an association between age and sex for the first spline coefficient in all three models, with different rates of bodyweight change between male and female cats. In male cats, the EMMs for bodyweight increased from 5.19 kg (95% CI 4.98, 5.40) at age 7 to 5.38 kg (95% CI 5.18, 5.57) at age 10.5 before decreasing to 4.93 kg (95% CI 4.64, 5.23) by age 16. In female cats, the association was negative between 7 and 10 years and more sharply negative thereafter, with EMMs decreasing from 4.35 kg (95% CI 4.17, 4.54) at age 7 to 4.28 kg (95% CI 4.10, 4.45) at age 10 and 3.67 kg (95% CI 3.40, 3.94) by age 16 (Figure 2B). On average, male cats were 0.83 kg heavier than female cats at baseline (the youngest age; 95% CI 0.29–0.96, FDR adjusted *p* < 0.001; Table 3; Figure 2B). In model 2, bodyweight was also negatively associated with the health status of the cat throughout the study period (Table 3), although there was also an interaction between age and health status with the first-degree spline term for age; in this respect, cats that remained healthy tended to be heavier than cats that developed morbidity or mortality as they aged (Figure 2C). Besides overall health status, there was a similar association between age and sex as that described for model 1 (Figure 2C). In model 3, cats with CKD, hyperthyroidism and neoplasia were lighter on average than cats without these conditions by 0.21 kg, 0.40 kg and 0.40 kg, respectively (Table 3), whilst cats with OE abnormalities were heavier on average by 0.16 kg than cats without OE abnormalities (Table 3).

### 3.3 Body condition score

**Figure 3:**
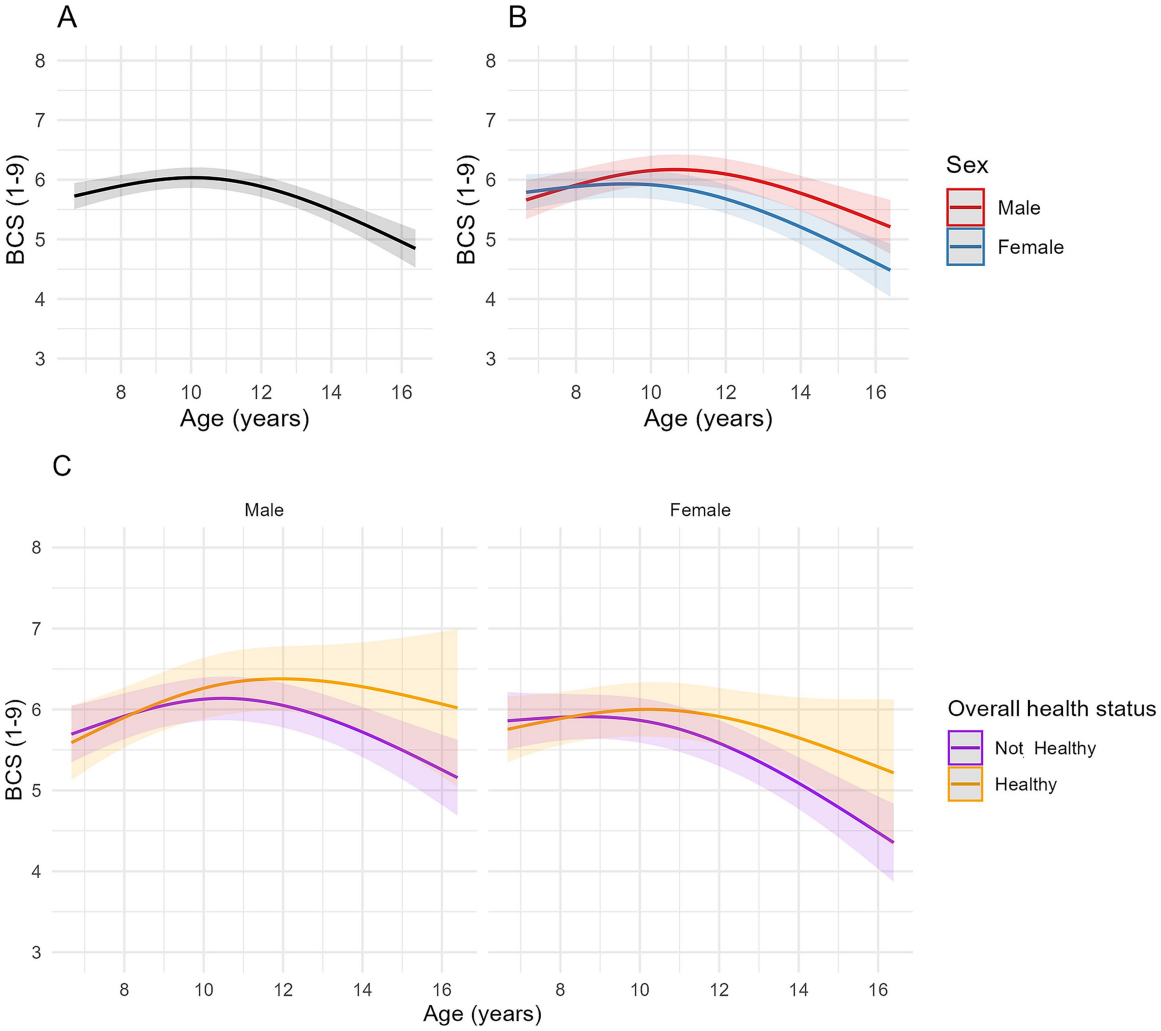
Longitudinal changes in body condition score (BCS) in cats enrolled on the Cat Prospective Ageing and Welfare Study. **(A,B)** Show the outcome of linear mixed-effects models with fixed effects of age (modelled with natural cubic splines for age in two degrees of freedom to account for non-linear associations of BCS and age), sex and the interactions of age and sex from 1,220 veterinary examinations from 209 cats, whilst **(C)** also included the overall health status of the cat over the course of the study and its interactions with age. The individual cat was added as a random effect. **(A)** Overall changes in BCS for all cats; **(B)** Overall changes in BCS for all cats separated by sex; **(C)** Overall changes in BCS in cats separated by sex (male cats shown in the left side plot, female cats in the plot on the right side of the figure) and whether the cat remained healthy over the study period or developed chronic morbidities and/or died (classed as “Not healthy”).

Data on BCS were available from 1,220 examinations from 209 cats (Table 1), with the median BCS at enrolment being 6 (IQR 5–7). Spaghetti plots, displaying longitudinal trends in BCS, are shown in the Supplementary Figure 3. The outcomes of the three linear mixed-effects models investigating changes in BCS with age in the cats are shown in Table 4 and Figure 3. As with bodyweight, a substantial amount of variation in BCS was due to differences amongst individual cats, with an ICC of 0.69–0.70. All three models fitted moderately well, with 70–72% of the variance in BCS being explained by a combination of the fixed and random effects.

**Table 4:** Outcomes of linear mixed-effects models with natural cubic splines for age examining the effects of age, sex, overall health status, and morbidities on body condition score in 209 cats enrolled on the Cat Prospective Ageing and Welfare Study.

Predictors	BCS [model 1]				BCS [model 2]				BCS [model 3]			
	Estimates	CI	Raw *p*-value	FDR adjusted *p*-value	Estimates	CI	Raw *p*-value	FDR adjusted *p*-value	Estimates	CI	Raw *p*-value	FDR adjusted *p*-value
(Intercept)	5.79	5.49–6.09	**<0.001**	**<0.001**	5.75	5.35–6.16	**<0.001**	**<0.001**	5.69	5.35–6.03	**<0.001**	**<0.001**
Age (years) [1st degree]	−0.44	−0.92 – 0.04	0.071	0.085	0.15	−0.54 – 0.84	0.672	0.672	−0.38	−0.91 – 0.15	0.160	0.201
Age (years) [2nd degree]	−1.45	−1.86 – −1.03	**<0.001**	**<0.001**	−0.72	−1.62 – 0.18	0.115	0.173	−1.01	−1.47 – −0.55	**<0.001**	**<0.001**
Sex [Male v Female at baseline]	−0.13	−0.57 – 0.31	0.563	0.563	−0.16	−0.61 – 0.28	0.465	0.598	−0.19	−0.63 – 0.26	0.405	0.434
Age (years) [1st degree] x Sex [Male]	1.06	0.36–1.76	**0.003**	**0.006**	1.22	0.50–1.93	**0.001**	**0.004**	1.33	0.60–2.07	**<0.001**	**0.001**
Age (years) [2nd degree] x Sex [Male]	0.65	0.07–1.24	**0.029**	**0.043**	0.73	0.14–1.31	**0.015**	**0.045**	0.57	−0.05 – 1.19	0.073	0.109
Health status over study [Not Healthy vs. Healthy at baseline]					0.10	−0.36 – 0.57	0.658	0.672				
Age (years) [1st degree] x Health status over study [Not Healthy]					−0.90	−1.68 – −0.11	**0.026**	0.058				
Age (years) [2nd degree] x Health status over study [Not Healthy]					−0.85	−1.79 – 0.09	0.076	0.137				
CKD									−0.34	−0.67 – 0.00	0.051	0.109
Hyperthyroidism									−0.66	−0.98 – −0.34	**<0.001**	**<0.001**
Hypertension									−0.05	−0.24 – 0.14	0.594	0.594
Neoplasia									−1.82	−2.39 – −1.25	**<0.001**	**<0.001**
Heart murmur									−0.12	−0.26 – 0.01	0.067	0.109
Diabetes mellitus									−0.40	−1.10 – 0.30	0.260	0.300
Dental disease									0.10	−0.01 – 0.22	0.069	0.109
OE Abnormalities [Unable to perform OE]									0.15	−0.04 – 0.34	0.123	0.168
OE Abnormalities [Yes]									0.18	0.01–0.36	**0.043**	0.108
	Random effects											
σ^2^	0.59				0.59				0.56			
τ_00_	1.32 _Cat_ID_				1.33 _Cat_ID_				1.29 _Cat_ID_			
ICC	0.69				0.69				0.70			
*N*	209 _Cat_ID_				209 _Cat_ID_				203 _Cat_ID_			
Observations	1,220				1,220				1,052			
Marginal *R*^2^/Conditional *R*^2^	0.032/0.700				0.039/0.705				0.067/0.718			

Age (years) 1st and 2nd degree relate to the 1st and 2nd degrees of freedom for a natural cubic spline model considering non-linear associations of age. Health status over study relates to whether the cat aged “healthily” or “not healthily” over the study period, i.e., whether the cat had developed morbidity or mortality by the end of the study period. P-values were adjusted for false discovery rate using the Benjamini-Hochberg method. BCS, body condition score, assessed using a 9-unit scale (14); CI, 95% confidence intervals; CKD, chronic kidney disease; OE, orthopaedic examination; ICC, intraclass correlation; FDR, false discovery rate. Values in bold indicate raw and FDR adjusted *p*-values of <0.05.

A negative association between age and BCS was identified for the second-degree spline term in models 1 and 3 (FDR adjusted *p* < 0.001), indicating that associations were non-linear, being positive until approximately 10.5 years, decreasing thereafter (Figure 3A). Whilst individual spline coefficients are not directly interpretable due to the nature of spline basis functions, the plotted trajectory reflects the overall effect of age on BCS. Estimated marginal means for BCS in model 1 increased from 5.77 (95% CI 5.56, 5.98) at age 7 to 6.03 (95% CI 5.86, 6.21) at age 10, then decreased to 4.96 (95% CI 4.66, 5.25) by age 16 (Figure 3A). There were no differences in BCS between male and female cats at younger ages, but the rate of change in BCS differed between male and female cats (Table 4; Figure 3B); in male cats, the association was positive until 11 years, and then negative thereafter whilst, in female cats, the association was flat until either 10 years (healthy ageing cats) or 9 years (unhealthy cats) before becoming negative (Figures 3B,C). In model 3, hyperthyroidism and neoplasia were negatively associated with BCS (Table 4).

**Figure 4:**
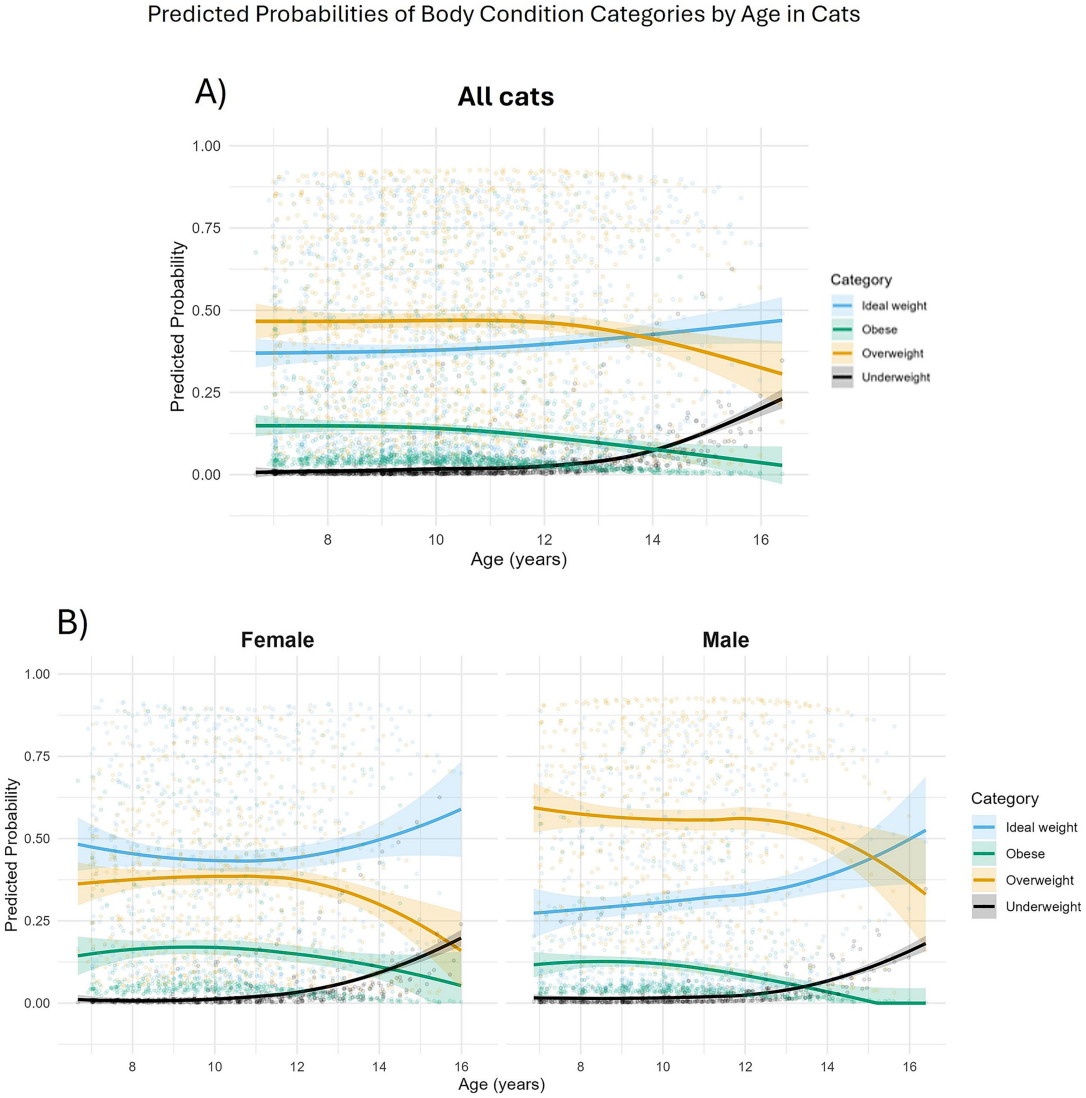
Changes in the predicted probabilities of body condition score categories with age in 209 cats enrolled on the Cat Prospective Ageing and Welfare Study. BCS group: “Obese” = BCS 8–9/9 (*N* = 156 observations from 51 cats); “Overweight” = BCS 6–7/9 (*N* = 559 from 150 cats); “Underweight” = 1–3/9 (*N* = 32 observations from 20 cats); “Ideal weight” = BCS 4–5/9 (*N* = 473 observations from 129 cats). **(A)** All cats; **(B)** Separated by sex (female cats in the left hand side figure, male cats in the right hand side figure).

When BCS was grouped into categories (14), there were 32 underweight (BCS 1–3/9) observations from 20 cats, 473 ideal weight (BCS 4–5/9) observations from 129 cats, 559 overweight (BCS 6–7/9) observations from 150 cats, and 156 obese (BCS 8–9/9) observations from 51 cats. The association between BCS category and age was again non-linear (Table 5; Figure 4). The probability of a cat having either an obese or overweight condition was relatively stable until approximately 10 years and 12 years, respectively. The probability of an obese or overweight body condition then decreased from 14.5% and 47.0% to 0.4% and 33.2%, respectively, by age 16 (Figure 4). The probability of a cat having an underweight condition remained relatively stable until approximately 12 years but increased thereafter from 2.7% to 13.6% by age 16 (Figure 4). By approximately 14 years of age, there was a greater predicted probability for a cat having a BCS 4–5/9 than BCS 6–7/9, and a greater probability of cats having a BCS of <3/9 compared with 8–9/9 (Figure 4). Further, the odds of a male cat being in overweight condition were 2.8 times greater than for a female cat (FDR adjusted *p* = 0.005; Table 5; Figure 4B).

**Table 5:** Results of a multinomial mixed-effect model examining associations of age and sex with body condition categories in 209 cats enrolled on the Cat Prospective Ageing and Welfare Study.

Predictors	Underweight vs. ideal weight				Overweight vs. ideal weight				Obese vs. ideal weight			
	Odds ratios	CI	Raw *p*-value	FDR adjusted *p*-value	Odds ratios	CI	Raw *p*-value	FDR adjusted *p*-value	Odds ratios	CI	Raw *p*-value	FDR adjusted *p*-value
(Intercept)	0.17	0.06–0.45	**<0.001**	**0.002**	0.87	0.42–1.80	0.705	0.846	0.02	0.00–0.12	**<0.001**	**<0.001**
Age (1st degree)	0.66	0.12–3.48	0.622	0.846	1.22	0.39–3.86	0.733	0.846	15.08	0.41–558.22	0.141	0.301
Age (2nd degree)	0.04	0.01–0.24	**0.001**	**0.002**	0.29	0.10–0.85	**0.024**	0.060	23.84	4.79–118.72	**<0.001**	**0.001**
Sex (Male vs. Female)	1.30	0.59–2.87	0.522	0.782	2.75	1.47–5.14	**0.002**	**0.005**	1.71	0.67–4.36	0.264	0.440
Health status over study (Not healthy vs. Healthy)	1.05	0.44–2.52	0.906	0.906	0.63	0.32–1.23	0.175	0.328	0.87	0.27–2.84	0.820	0.879
N _Cat_ID_							209					
Observations							1,220					

Age (years) 1st and 2nd degree relate to the 1st and 2nd degrees of freedom for a natural cubic spline model considering non-linear associations of age. Health status over study relates to whether the cat aged “healthily” or “not healthily” over the study period, i.e., whether the cat had developed morbidity or mortality by the end of the study period. Underweight (BCS 1–3/9; *N* = 32 observations from 20 cats), ideal weight (BCS 4–5/9; *N* = 473 observations from 129 cats; reference category), overweight (BCS 6–7; N = 559 from 150 cats) and obese (BCS 8–9; *N* = 156 observations from 51 cats). P-values were adjusted for false discovery rate (FDR) using the Benjamini-Hochberg method. Values in bold indicate raw and FDR adjusted *p*-values of <0.05.

### 3.4 Muscle condition score

**Figure 5:**
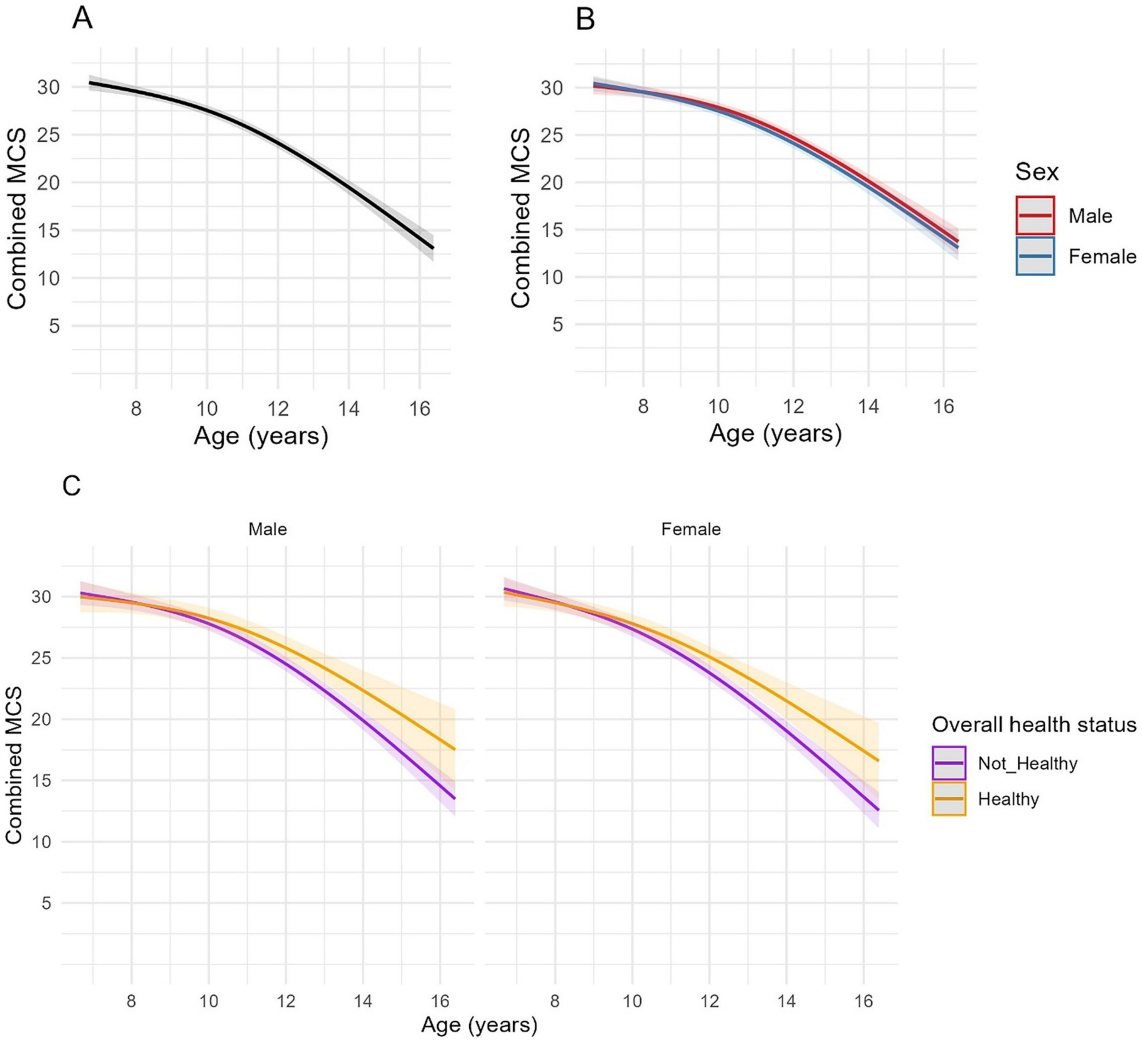
Longitudinal changes in combined muscle condition score (MCS) in cats enrolled on the Cat Prospective Ageing and Welfare Study. Combined MCS relates to a combined score for 10 skeletal landmarks each graded 0 (severe muscle loss) to 3 [no muscle loss (15);], to a total of 30 points. **(A,B)** Show the outcome of linear mixed-effects models with fixed effects of age (modelled with natural cubic splines for age in two degrees of freedom to account for non-linear associations of combined MCS and age), sex and the interactions of age and sex from 1,144 veterinary examinations from 208 cats, whilst **(C)** also included the overall health status of the cat over the course of the study and its interactions with age. The individual cat was added as a random effect. **(A)** Overall changes in MCS for all cats; **(B)** Overall changes in MCS for all cats separated by sex; **(C)** Overall changes in MCS in cats separated by sex (male cats shown in the left side plot, female cats in the right-side plot) and whether the cat remained healthy over the study period or developed chronic morbidities and/or died (classed as “Not healthy”).

Muscle condition scores were available from 1,144 clinical examinations of 208 cats, with a median of 5 assessments per cat (IQR 2–8, range 1–12). The median combined MCS at enrolment was 30 (IQR 28–30). Spaghetti plots displaying longitudinal trends in combined MCS within cats are provided in the Supplementary Figure 4. Based on ICC, 34–38% of the variance in combined MCS was due to differences amongst cats (Table 6), whilst the fixed and random effects in the three models explained ~69–70% of the variance in the data. Combined MCS decreased with age, in a non-linear fashion, whereby the association was more strongly negative after 10 years (Figure 5). The EMMs for model 1 showed MCS decreased gradually from 30.0 points to 27.7 points between ages 7 and 10, before decreasing at a greater rate to 14.5 points by age 16 (Figure 5A). In contrast to bodyweight and BCS, no differences were evident between sexes (Figure 5B; Table 6). However, there was a negative association between combined MCS, age and overall health status, whereby the age-associated decrease in combined MCS was steeper after 10 years for cats categorised as ‘not healthy’ during the study period (Figure 5C; Table 6). Further, combined MCS was less in cats diagnosed with hyperthyroidism, neoplasia and heart murmurs than for cats without these conditions by an average of 3.2, 7.0, and 0.8 points, respectively, (FDR adjusted *p* ≤ 0.001 for all; Table 6).

**Table 6:** Outcomes of linear mixed-effects models with natural cubic splines for age examining the effects of age, sex, overall health status, and morbidities on combined muscle condition score in 208 cats enrolled on the Cat Prospective Ageing and Welfare Study.

Predictors	MCS [model 1]				MCS [model 2]				MCS [model 3]			
	Estimates	CI	Raw *p*-value	FDR adjusted *p*-value	Estimates	CI	Raw *p*-value	FDR adjusted *p*-value	Estimates	CI	Raw *p*-value	FDR adjusted *p*-value
(Intercept)	30.44	29.62–31.26	**<0.001**	**<0.001**	30.35	29.21–31.49	**<0.001**	**<0.001**	30.72	29.77–31.67	**<0.001**	**<0.001**
Age (years) [1st degree]	−13.69	−15.33 – −12.06	**<0.001**	**<0.001**	−11.27	−13.67 – −8.87	**<0.001**	**<0.001**	−11.82	−13.57 – −10.07	**<0.001**	**<0.001**
Age (years) [2nd degree]	−16.20	−17.63 – −14.77	**<0.001**	**<0.001**	−12.68	−15.91 – −9.45	**<0.001**	**<0.001**	−13.96	−15.52 – −12.40	**<0.001**	**<0.001**
Sex [Male v Female at baseline]	−0.26	−1.47 – 0.96	0.680	0.680	−0.36	−1.58 – 0.86	0.562	0.632	−0.28	−1.48 – 0.91	0.644	0.795
Age (years) [1st degree] x Sex [Male]	1.51	−0.89 – 3.90	0.217	0.325	2.02	−0.39 – 4.44	0.101	0.151	1.96	−0.49 – 4.40	0.116	0.201
Age (years) [2nd degree] x Sex [Male]	0.53	−1.49 – 2.55	0.607	0.680	0.81	−1.22 – 2.84	0.433	0.556	0.35	−1.75 – 2.46	0.742	0.795
Health status over study [Not Healthy vs Healthy at baseline]					0.31	−0.98 – 1.60	0.637	0.637				
Age (years) [1st degree] x Health status over study [Not Healthy]					−3.47	−6.16 – −0.78	**0.011**	**0.026**				
Age (years) [2nd degree] x Health status over study [Not Healthy]					−4.02	−7.37 – −0.67	**0.019**	**0.034**				
CKD									−0.24	−1.18 – 0.71	0.623	0.795
Hyperthyroidism									−3.19	−4.16 – −2.23	**<0.001**	**<0.001**
Hypertension									−0.50	−1.13 – 0.13	0.120	0.201
Neoplasia									−6.98	−8.88 – −5.07	**<0.001**	**<0.001**
Heart murmur									−0.80	−1.24 – −0.36	**<0.001**	**0.001**
Diabetes mellitus									−0.84	−3.14 – 1.46	0.474	0.711
Dental disease									0.01	−0.37 – 0.39	0.972	0.972
OE abnormalities [Unable to perform OE]									−0.12	−0.75 – 0.51	0.710	0.795
OE Abnormalities [Yes]									−0.47	−1.07 – 0.12	0.118	0.201
	Random effects											
σ^2^	7.16				7.11				6.80			
τ_00_	4.43 _Cat_ID_				4.39 _Cat_ID_				3.50 _Cat_ID_			
ICC	0.38				0.38				0.34			
N	208 _Cat_ID_				208 _Cat_ID_				202 _Cat_ID_			
Observations	1,144				1,144				1,013			
Marginal *R*^2^/Conditional *R*^2^	0.500/0.691				0.510/0.697				0.545/0.700			

Age (years) 1st and 2nd degree relate to the 1st and 2nd degrees of freedom for a natural cubic spline model considering non-linear associations of age. Health status over study relates to whether the cat aged “healthily” or “not healthily” over the study period, i.e., whether the cat had developed morbidity or mortality by the end of the study period. MCS, muscle condition score, relates to a combined score for 10 skeletal landmarks each graded 0 (severe muscle loss) to 3 (no muscle loss (15);) to a total of 30 points; CI, 95% confidence intervals. *p*-values were adjusted for false discovery rate using the Benjamini-Hochberg method. CKD, chronic kidney disease; OE, orthopaedic examination; ICC, intraclass correlation; FDR, false discovery rate. Values in bold indicate raw and FDR adjusted *p*-values of <0.05.

**Figure 6:**
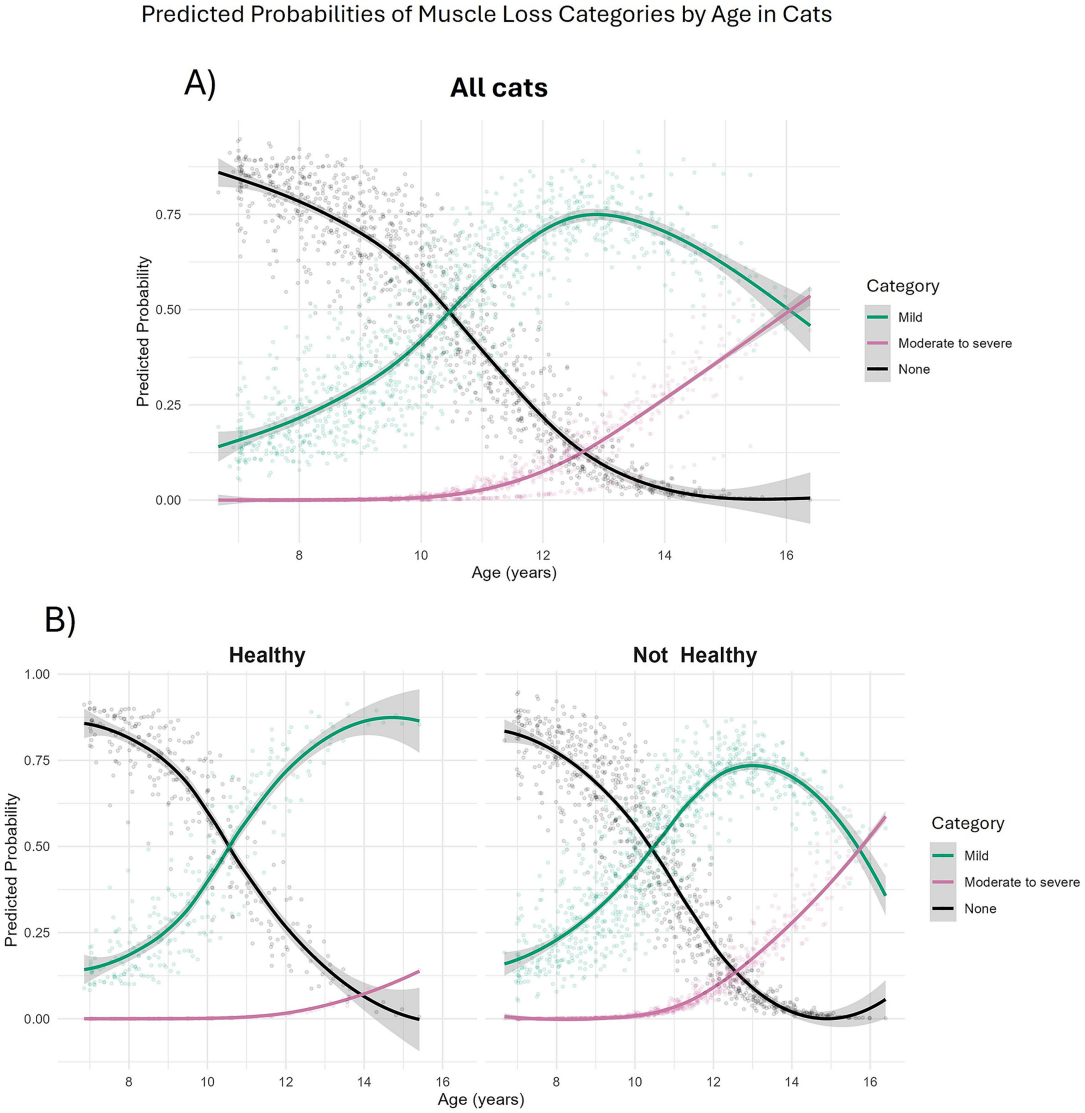
Predicted probabilities of muscle condition score categories in 208 cats enrolled on the Cat Prospective Ageing and Welfare Study. Predicted probabilities were extracted from a multinomial mixed effects model investigating effects of age, sex and overall health status of the cat over the study period on categories of muscle condition scores. A combined muscle condition score was taken from 10 skeletal landmarks each score from 3 (no muscle loss) to 0 (severe muscle loss). Muscle condition scores were then categorised as none (combined score of 30/30, N = 540 observations from 177 cats); Mild (combined score of 20–29/30, N = 556 observations from 164 cats); Moderate to Severe (combined score of 0–19/30, N = 68 observations from 44 cats). **(A)** All cats; **(B)** Separated by health status over study. “Healthy” (left side) relates to cats that aged healthily, “Not healthy” (right side) related to cats that had developed morbidity or mortality by the end of the study period.

Muscle loss was also categorised into mild (MCS 20–29/30; 556 observations from 164 cats), moderate-to-severe (MCS 0–19/30; 68 observations from 44 cats) or no (MCS 30/30; 540 observations from 177 cats) muscle loss and, again, age was associated with severity. Compared with cats having no muscle loss, the odds of a cat having muscle loss increased with age in a non-linear fashion (Table 7). The predicted probability of a cat having mild muscle loss increased from 16.3% at age 7, to 41.5% at age 10 and surpassed the probability of no muscle loss at 10.5 years (Figure 6A). The predicted probability of a cat having moderate-to-severe muscle loss increased from 0.0% at age 7 to 48.4% by age 16 and surpassed that of having no muscle loss at ~13 years and mild muscle loss at ~16 years (Figure 6A). Cats classified as not ageing healthily had 8 times the odds of having moderate-to-severe muscle loss compared with those classified as ageing healthily (FDR adjusted *p* = 0.011; Table 7). In both healthy and not healthy ageing cats over age 10.5 years, the predicted probability of mild muscle wastage exceeded that of no muscle wastage (Figure 6B). The probability of moderate-to-severe muscle loss exceeded that of mild muscle loss after age 16 years only in cats not ageing healthily; in healthy ageing cats the probability of moderate to severe muscle loss did not exceed that of mild muscle loss across the age range studied (Figure 6B).

**Table 7:** Results of a multinomial mixed-effects model examining changes in grade of muscle wastage with increasing age, sex and whether the cat remained healthy over the study period in 208 cats enrolled on the Cat Prospective Ageing and Welfare Study.

Predictors	Mild vs none				Moderate to severe vs none			
	Odds ratios	CI	Raw *p*-value	FDR adjusted *p*-value	Odds Ratios	CI	Raw *p*-value	FDR adjusted *p*-value
(Intercept)	0.14	0.08–0.26	**<0.001**	**<0.001**	0.00	0.00–0.00	**<0.001**	**<0.001**
Age (1st degree)	1.09e+03	3.72e+02–3.19e+03	**<0.001**	**<0.001**	6.65e+07	1.10e+04–4.00e+11	**<0.001**	**<0.001**
Age (2nd degree)	0.72	3.35e+02–9.55e+03	**<0.001**	**<0.001**	6.61e+05	4.71e+04–9.28e+06	**<0.001**	**<0.001**
Sex (Male vs Female)	1.21	0.49–1.04	0.081	0.101	0.66	0.34–1.30	0.231	0.257
Health status over study (Not healthy vs Healthy)	1.21	0.80–1.83	0.364	0.364	8.04	1.74–37.22	**0.008**	**0.011**
N _Cat_ID_		208						
Observations		1,144						

Age (years) 1st and 2nd degree relate to the 1st and 2nd degrees of freedom for a natural cubic spline model considering non-linear associations of age. Health status over study relates to whether the cat aged “healthily” or “not healthily” over the study period, i.e., whether the cat had developed morbidity or mortality by the end of the study period. A combined muscle condition score was taken from 10 skeletal landmarks each score from 3 (no muscle loss) to 0 (severe muscle loss). Muscle wastage was then graded as none (combined score of 30/30, *N* = 540 observations from 177 cats, reference category in model); Mild (combined score of 20–29/30, *N* = 556 observations from 164 cats); Moderate to Severe (combined score of 0–19/30, *N* = 68 observations from 44 cats). P-values were adjusted for false discovery rate (FDR) using the Benjamini-Hochberg method. Values in bold indicate raw and FDR adjusted *p*-values of <0.05.

## 4 Discussion

In this prospective cohort study of ageing in cats, we have analysed age-related changes in three body composition metrics (bodyweight, BCS and combined MCS), and assessed associations with sex and health status. For all three metrics, the association with age was non-linear; bodyweight and BCS both slightly increased over middle age on average, before decreasing after age 10 years. There was a decrease in combined MCS during middle age, but the rate of decrease was greater after around 10 years of age. These findings concur with a previous study examining changes in bodyweight, BCS and body fat and lean tissue content measured by dual energy X-ray absorption [DEXA; (22)]. Although sex differences were not examined in that study, cats on average gained bodyweight until 9 years, then lost bodyweight thereafter. Body condition score also increased until ~11 years of age, before decreasing, and cats had a substantial decline in lean tissue mass after 12 years of age (22). Similar trajectories, to those noted for the cats in this study, have also been reported in human body composition studies (42, 43). For example, fat-free mass decline occurs from 47 years in people (44), equivalent to ~8 years of age in cats, which is similar to the decrease in muscle condition observed in the cats in this study. Body fat in humans generally increases during middle age, before decreasing in older age (43). This middle-age-related increase in body fat is thought to result from various factors, including decreased resting metabolic rate and decreased fat oxidation (45). The reasons for the decline in bodyweight, muscle mass and body fat in elderly people are complex, with a loss of the ability to maintain energy balance thought to be most likely (10, 46, 47). An age-related decline in muscle mass is increasingly considered to be an important predictor of health in both human and veterinary medicine, not least because it contributes to frailty and affects quality of life (10).

Categories of BCS (e.g., underweight, ideal weight and overweight-to-obese body condition) were relatively stable during middle age in the cats of the current study. Cats were most likely to be in overweight condition during their ‘mature’ life stage (age 7 to 10 years), a finding that agrees with the results of a recent cross-sectional study of electronic health records from cats in veterinary hospitals in the United States (48). There was a subsequent cross-over in body condition, with more cats being classed as underweight than obese, and more being classified as ideal weight than overweight after 14 years of age. The age when more cats were predicted to have mild muscle loss than no muscle loss (~10 years) also concurs with the point when cats are considered to have entered ‘old age’ (3).

There were differences between male and female cats in how changes in bodyweight and BCS evolved over time; male cats were heavier, on average, than female cats, and gained more bodyweight and body condition during middle age, before losing bodyweight and body condition after 10 years and 12 years of age, respectively; in contrast, female cats initially lost weight gradually during middle age, but then more rapidly when older than 10 years, whilst body condition initially remained stable before decreasing after 9 years. When body condition was categorised from underweight to obese, male cats were 2.8 times more likely to have an overweight condition than female cats, a finding that has been observed in previous studies (18, 49, 50). Neutered female cats have a greater resting metabolic rate than neutered male cats (51) and, therefore, males might be more prone to consuming excess energy and gaining weight. Given that male cats tend to weigh more than female cats, it has also been hypothesised that there may be some confirmation bias when observers perform a BCS on a heavier cat (52, 53). It is unclear whether this difference in body condition between sexes accounts for the increased odds of male cats developing diabetes mellitus (54–56). Further research is required to investigate the reasons for this increased risk in male cats, and any related disease associations.

In contrast to BCS, no sex differences were evident for combined MCS and muscle loss in the current study. In humans, males tend to have a greater percentage of lean tissue mass than females, and both sexes tend to gain body fat during middle age, with men accumulating more visceral fat, and women more subcutaneous fat (57). Menopause in women leads to a shift in fat distribution with a subsequent increase in visceral fat (58, 59). All but two cats in this study were neutered and, therefore, the effect of sex hormones with age will not be as impactful as in human studies. An accumulation of body fat and decrease in lean tissue can occur in cats after neutering (51); however, no study to date has examined differences between body composition in neutered and sexually-intact cats throughout the whole lifespan.

Cats that were diagnosed with a chronic disease, or died during the study period, lost bodyweight at a greater rate than those that remained healthy. Specifically, cats with hyperthyroidism and neoplasia were associated with a decreased BCS, whilst hyperthyroidism, neoplasia and CKD were associated with an overall decreased bodyweight. Hyperthyroidism, neoplasia and heart murmur presence were also associated with decreased MCS. Loss of bodyweight, BCS and MCS have previously been documented in hyperthyroidism, and are thought to be the result of effects of thyroxine on metabolism (12). An association between cardiac disease and muscle loss in cats has previously been reported (60), whilst MCS is better able to identify cachexia in cats with congestive heart failure, compared with BCS (13). Bodyweight was greater in cats with OE abnormalities, a finding that concurs with previous findings whereby the odds of having a musculoskeletal condition were greater in cats with a BCS of >6/9 (61).

The longitudinal findings outlined in this study have implications for clinical veterinary practice. Understanding the typical trajectories of bodyweight, BCS and muscle condition can improve the identification of individuals that deviate from the expected age-related patterns and therefore may be at greater risk of certain age-related diseases. For example, loss of bodyweight and BCS before the age of 11 years in male cats and 10 years in female cats, or accelerated loss of muscle condition before aged 10 years in either sex, could warrant further investigation of underlying disease or support early detection of sarcopenia and frailty. The findings also support the tailoring of nutritional interventions in mature and senior cats, particularly in order to maintain lean body mass (62). Additionally, considering proactive physiotherapy or lifestyle adjustments to encourage activity may prevent overweight and obesity in mature cats and preserve muscle mass in senior cats (63). Finally, awareness of typical trajectories of body and muscle condition should be considered when planning anaesthetics and pharmacological protocols, as decreased muscle mass may affect drug metabolism and thermoregulation (64, 65). Incorporating routine assessment of bodyweight, BCS and MCS during routine senior wellness examinations from middle age onwards in cats may support earlier intervention, preserve healthspan and improve outcomes in ageing cats.

Given that this was a cohort of pet cats, one limitation of the current study was the use of non-invasive body composition metrics, such as BCS and MCS, not least that these are subjective measurements, and less accurate than using bespoke objective techniques such as DEXA. That said, the 9-point BCS and scale used for the MCS correlate well with DEXA measurements of body fat mass (14, 66) and muscle mass (15), respectively, whilst MCS also correlates with epaxial muscle height as measured by ultrasonography (67). Further, all assessments were conducted by four trained veterinary professionals (one registered veterinary nurse and three veterinarians), which will likely have decreased subjectivity of these scores. A second limitation was the lack of definitive diagnosis of certain diseases, for example orthopaedic disease, given that detailed investigations (e.g., diagnostic imaging) were not undertaken in many of the cats. Therefore, we chose to use the term “OE abnormality” for such cats in the current study, rather than trying to classify according to the exact condition (e.g., osteoarthritis). Further categorising the level of OE abnormality could be useful, although this would have limited the available sample size. In a similar manner, the presence of both heart murmurs and hypertension were also recorded, but further investigations were often not performed to determine the exact nature of any underlying cardiac or other disease.

The cats in this study were from one geographical location (the North-West of England), and validation in more geographically diverse cat populations would allow the generalisability of the findings to be assessed. Furthermore, breed differences and the effect of clustering within owners were not accounted for in the present study. The majority of cats (86%) were non-pedigree (domestic shorthair, medium hair or long hair), with the remaining 14% representing multiple breeds that were too heterogenous and small in number to allow for meaningful statistical analysis. Future studies to explore potential breed-related differences in body composition changes with age would require larger cohorts with sufficient sample sizes of different breeds. As some cats died over the course of the study (59/209, 28%) or were lost to follow up (59/209, 28%) there is a risk of attrition bias if those cats differed systematically from those retained, which could have influenced the data at later ages. Future studies could utilise statistical techniques that incorporate both mixed-effect models and survival models, known as joint models (68). These could prove particularly useful in estimating, for example, how changes in bodyweight between veterinary examination alter the risk of death or disease.

A final limitation was the fact that the exact age of some of the cats (73/209, 35%) was not known because they had been acquired by their current owners as adults with an unknown date of birth. As a result, in some cases the age had to be estimated, which might have affected the accuracy of the results. Despite this limitation, data from these cats would still be valuable, not least because longitudinal changes could still be assessed.

## 5 Conclusion

Longitudinal changes in bodyweight, BCS and combined MCS have been investigated in a prospective cohort study of pet cats as they aged. There were non-linear changes over middle age and old age in cats, with decreases in bodyweight, BCS and combined MCS in older cats. These findings highlight the importance of recording body composition metrics within clinical examinations of ageing cats. They also indicate the need for an improvement in the maintenance of muscle mass in older cats, and to better understand the increased risk of overweight in male cats.

## Data Availability

The original contributions presented in the study are included in the article/Supplementary material, further inquiries can be directed to the corresponding author/s.
