# Supplementary material for: Longitudinal changes in bodyweight, body condition, and muscle condition in ageing pet cats: findings from the Cat Prospective Ageing and Welfare Study
Source: Front Vet Sci. 2025 Aug 25;12:1654002. doi: 10.3389/fvets.2025.1654002 (PMC12415779; doi:10.3389/fvets.2025.1654002)
Supplement: Supplementary file 1 [file Data_Sheet_1.docx]

Supplementary Material

# Supplementary Tables

**Supplementary Table 1. R code for the formulae used for linear mixed-effects models investigating age, sex and health related effects on body composition metrics in cats enrolled on the Cat Prospective Ageing and Welfare Study.**

| Model 1 | Model 2 | Model 3 |
| --- | --- | --- |
| lmer(bodyweight ~ ns(Age_years, df=2) + Sex + ns(Age_years, df=2):Sex +(1\|Cat_ID), data) | lmer(bodyweight ~ ns(Age_years, df=2) + Sex +ns(Age_years, df=2):Sex + Overall_health_status + ns(Age_years, df=2):Overall_health_status +(1\|Cat_ID),data) | lmer(bodyweight ~ ns(Age_years, df = 2) + Sex + ns(Age_years, df=2):Sex + CKD + Hyperthyroidism + Hypertension + Neoplasia + Heart_murmur + Diabetes + Dental_disease + OE_abnormalities+ (1 \| Cat_ID), data) |

Models were repeated separately for body condition score and muscle condition score as dependent variables instead of bodyweight. The packages in R used for these analyses were ‘lme4’ version 1.1-35.4 (Bates et al., 2015) and ‘splines’ (version 4.4.1; R Core Team 2024). Abbreviations: CKD= chronic kidney disease, OE=orthopaedic examination.

**Supplementary Table 2. A summary of the breeds of 209 cats enrolled onto the Cat Prospective Ageing and Welfare Study.**

| Breed | | N (%) |
| --- | --- | --- |
| Non Pedigree |  |  |
|  | DLH | 23 (11%) |
|  | DMH | 24 (11%) |
|  | DSH | 133 (64%) |
| Total |  | **180 (86%)** |
| Pedigree | **Pedigree** |  |
|  | American Exotic | 2 (1.0%) |
|  | Balinese | 1 (0.5%) |
|  | Bengal | 3 (1.4%) |
|  | British short hair | 3 (1.4%) |
|  | Egyptian Mau | 1 (0.5%) |
|  | Maine coon | 2 (1.0%) |
|  | Ocicat | 1 (0.5%) |
|  | Oriental | 2 (1.0%) |
|  | Persian | 4 (1.9%) |
|  | Ragdoll | 4 (1.9%) |
|  | Russian blue | 1 (0.5%) |
|  | Siamese | 3 (1.4%) |
|  | Somali | 1 (0.5%) |
|  | Sphynx | 1 (0.5%) |
| Total |  | **29 (14%)** |

*Abbreviations: DSH=Domestic shorthair, DMH=Domestic medium hair, DLH=Domestic long hair*

#
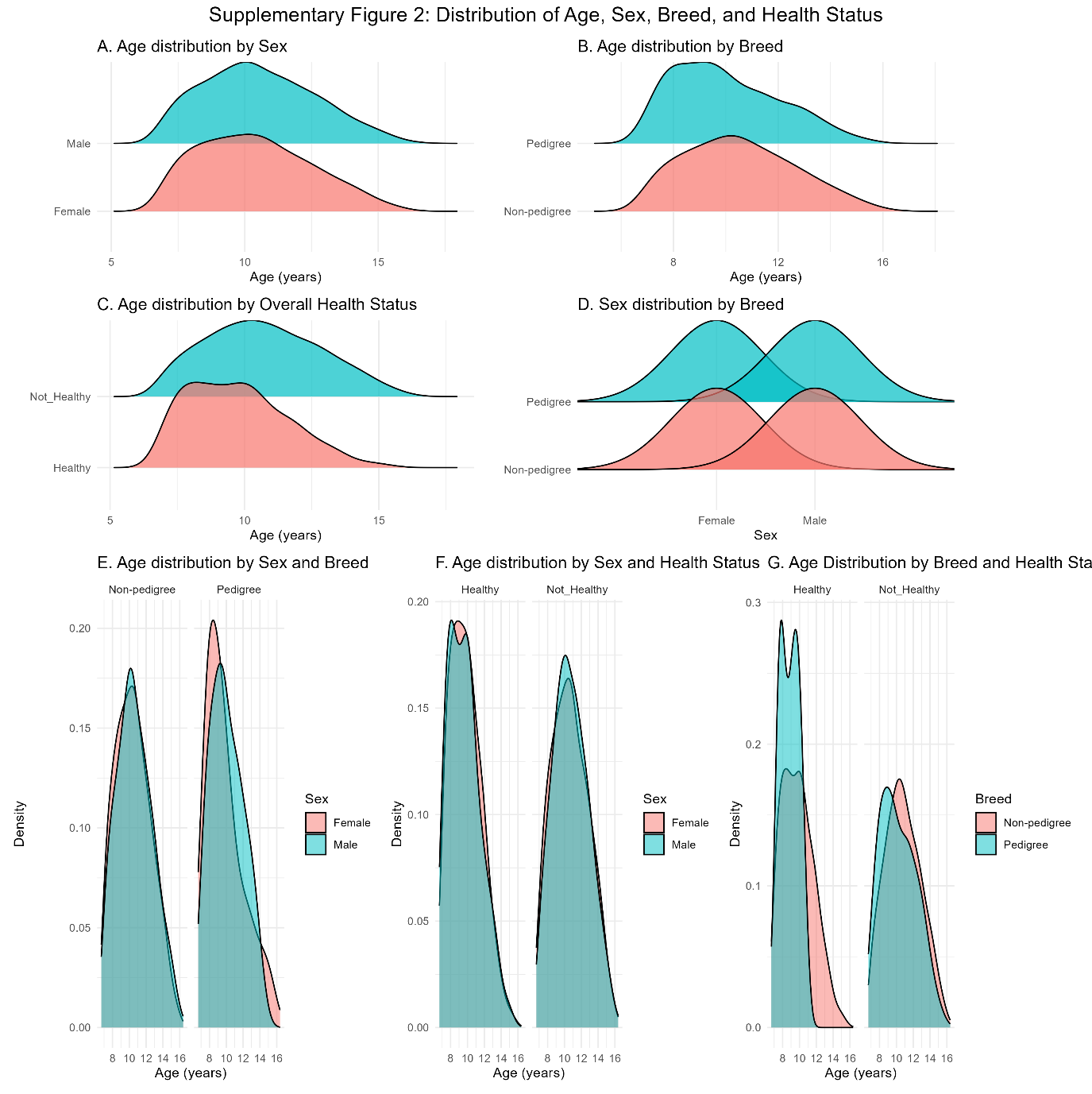
Supplementary Figures

**Supplementary Figure 1. Distribution of data by age, sex, breed, and health status in 209 cats enrolled in the Cat Prospective Ageing and Welfare Study**. Each panel illustrates the age distribution across covariates, with sample sizes reported as number of cats (N) and number of observations (Obs) in parentheses: (A) Age distribution by sex: Female (N = 111, Obs = 644) and Male (N = 98, Obs = 587). (B) Age distribution by breed: Non-pedigree (N = 180, Obs = 1104) and Pedigree (N = 29, Obs = 127). (C) Age distribution by overall health status: Healthy (N = 73, Obs = 321) and Not Healthy (N = 136, Obs = 910). (D) Distribution of sex across breed groups. (E) Age distribution stratified by sex within each breed group. (F) Age distribution stratified by sex within each overall health status group. (G) Age distribution stratified by breed within each overall health status group.


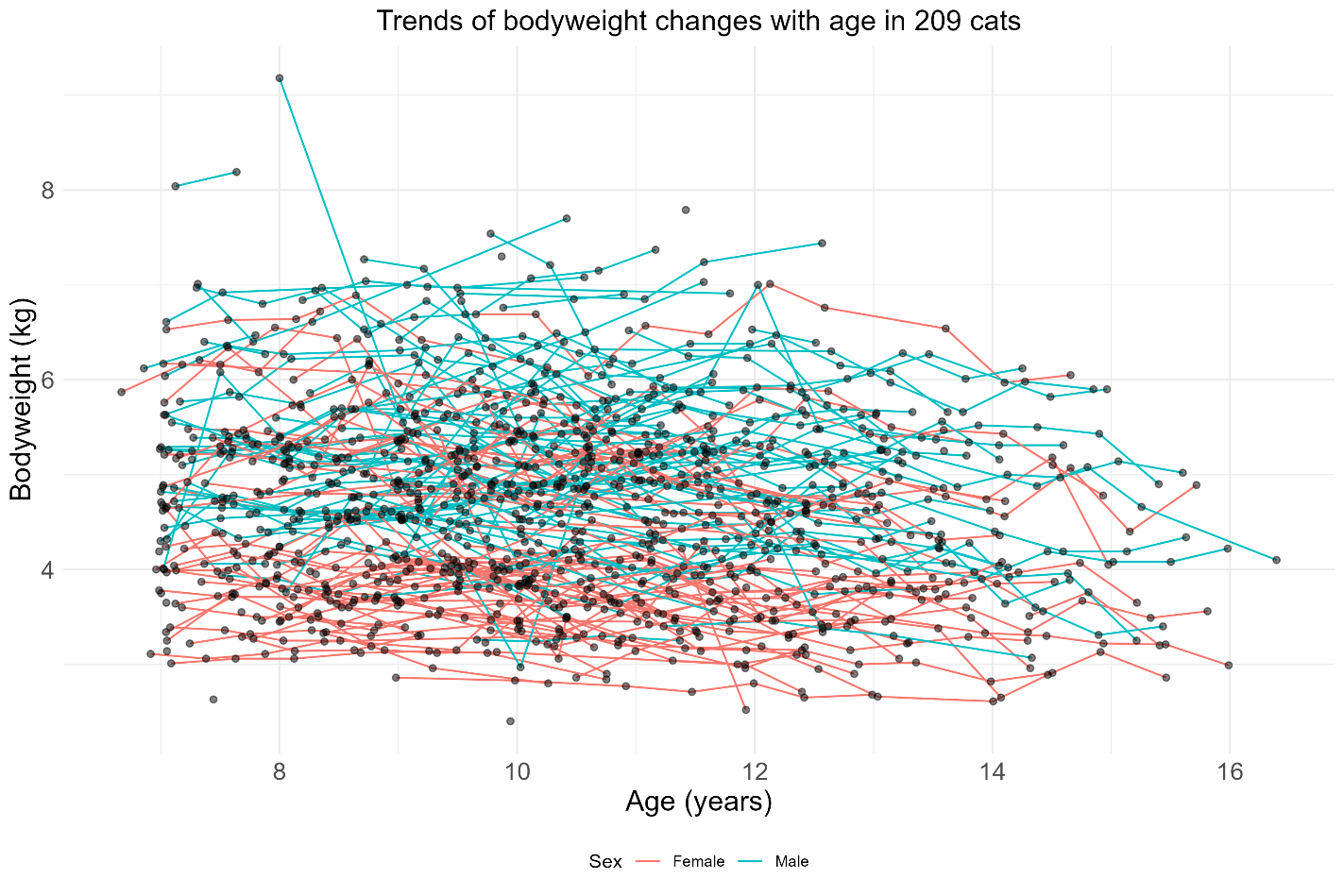


Supplementary Figure 2. Changes in bodyweight with increasing age in 209 cats enrolled in the Cat Prospective Ageing and Welfare Study. Data points are from a total of 1231 veterinary examinations. Each point on the graph represents a recorded bodyweight at a visit to the study. Lines link repeated measures over time in the same cat. Male cats are shown in blue (N=98) and female cats in red (N=111)


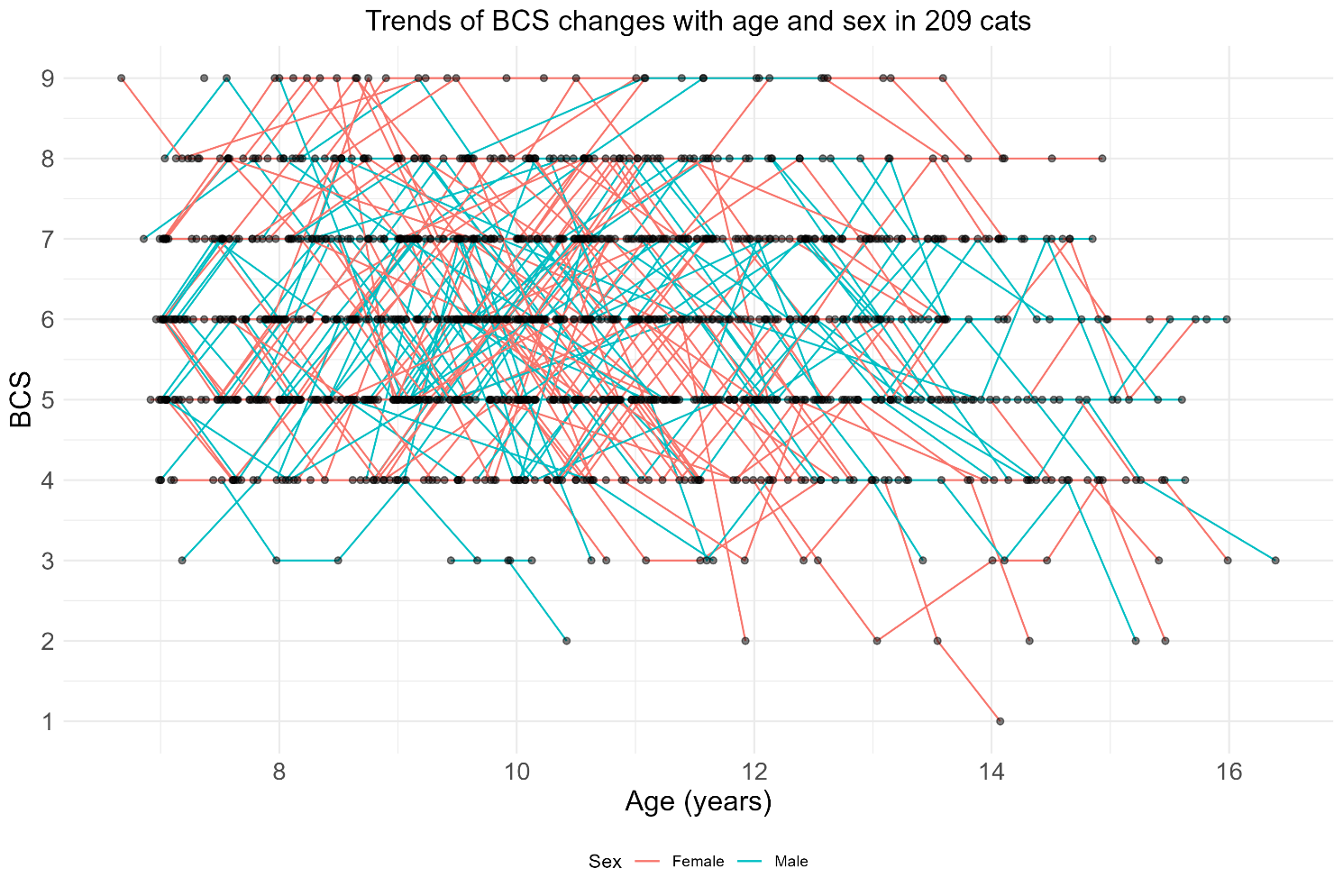


Supplementary Figure 3. Changes in body condition score (BCS) with age in 209 cats enrolled on the Cat Prospective Ageing and Welfare Study. Data points are from a total of 1220 veterinary examinations. Each point on the graph represents a recorded BCS at a visit to the study. Lines link repeated measures over time in the same cat. Male cats are shown in blue (N=98) and female cats in red (N=111).


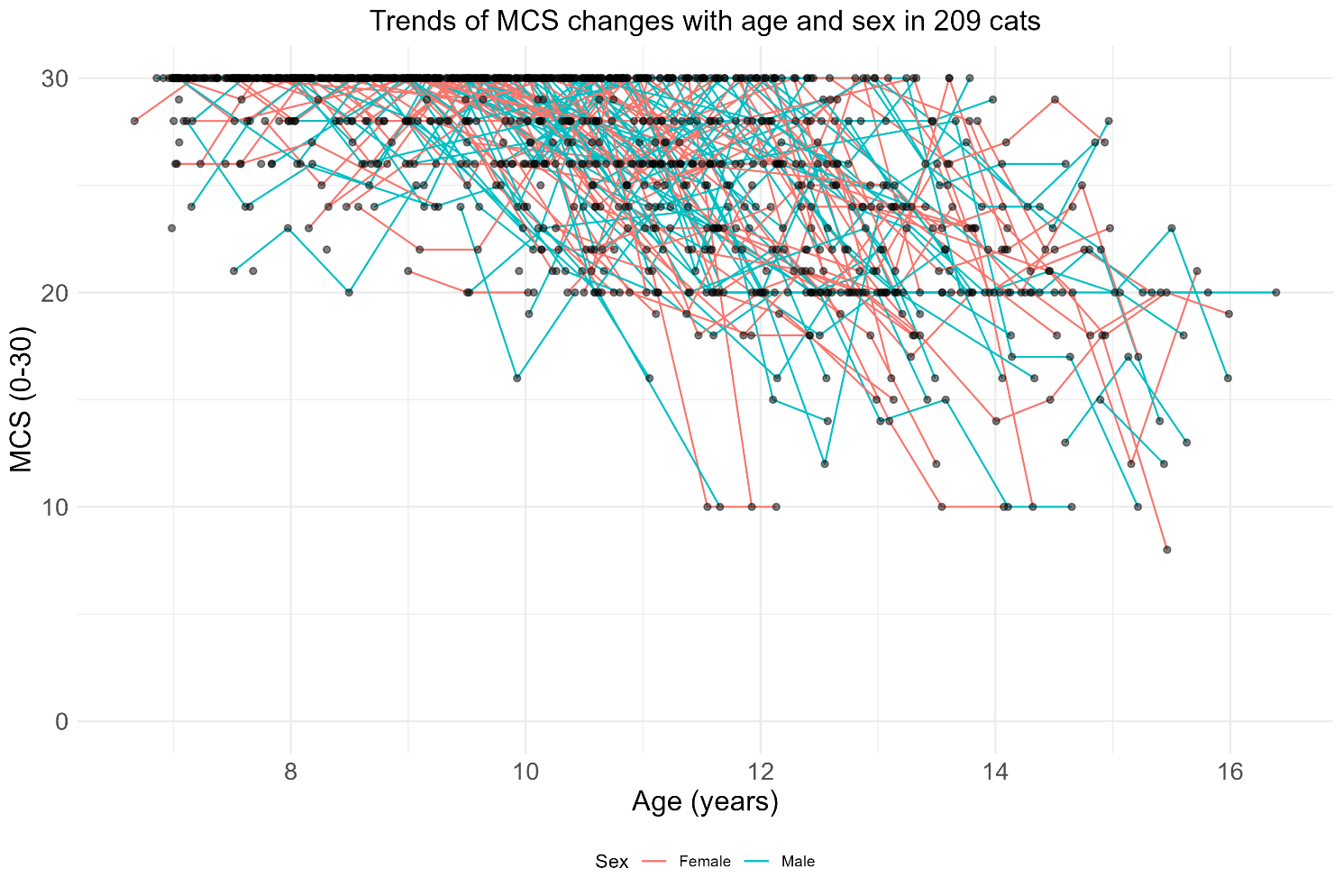


Supplementary Figure 4. Changes in combined muscle condition score (MCS) with increasing age in 208 cats enrolled in the Cat Prospective Ageing and Welfare Study. Data points are from a total of 1143 veterinary examinations. Data points on the graph represent a combined MCS taken from ten skeletal landmarks, each with a score of 0 (severe muscle loss) to 3 (no muscle loss), to give a possible combined MCS of between 0 and 30. Lines link repeated measures over time in the same cat. Male cats are shown in blue (N=98) and female cats in red (N=111).
